# Ultrasound‐Guided External Trigger Point Injections for Female Patients With Myofascial Pelvic Pain: A Report of Two Cases

**DOI:** 10.1155/cria/7853542

**Published:** 2025-11-14

**Authors:** Sophie E. Smith, Alopi Patel

**Affiliations:** ^1^ Department of Anesthesiology, Rutgers Robert Wood Johnson Medical School, New Brunswick, New Jersey, USA, rutgers.edu

**Keywords:** female pelvic floor dysfunction, high-tone pelvic floor disorder, hypertonicity, myofascial pelvic pain, ultrasound-guided trigger point injections

## Abstract

Trigger point injections (TPIs) are used to relieve myofascial pain via injection with local anesthetics. External TPIs are commonly used to treat surface muscle groups such as the trapezius, while internal transvaginal or external pelvic TPIs may be used for pelvic pain in female patients. This case series discusses two female patients with histories of myofascial pelvic pain due to high‐tone pelvic floor disorder (HTPFD) who received ultrasound‐guided external TPIs for pain relief. Patient 1 had previously received internal TPIs with no relief, while Patient 2 had previously tried botulinum toxin injections and a pudendal nerve block with some relief. In both cases, ultrasound‐guided external TPIs provided pain relief for a period of time and were repeated. This case highlights the importance of ultrasound‐guided external TPIs as part of the arsenal of treatment options for pelvic floor dysfunction treatment.

## 1. Introduction

A widely‐used clinical definition for chronic pelvic pain is noncyclical pelvic pain that lasts for at least 6 months [1]. As is with most chronic pain conditions, there are multifactorial contributors to the pain experience. Although a diagnosis of pelvic pain may be made, there are often a number of individual contributors such as myofascial components and central sensitization. High‐tone pelvic floor disorder (HTPFD), also known as myofascial pelvic pain syndrome, is one of the most common contributing causes of chronic pelvic pain in women [2]. HTPFD is characterized by areas of hypertonicity, or abnormally increased resting muscle tone or tightness, of the pelvic floor muscles [3–5].

Myofascial trigger points, which are localized areas of tenderness within a taut portion of skeletal muscle, may occur in hypertonic muscles [2, 6–8]. Trigger points may be a result of muscle injury, which may include a perceptible trauma or repeated microtrauma, or may occur without a clear inciting incident [7, 8]. Trigger points are best identified upon physical examination because they feel taut when palpated perpendicular to the fiber direction and are painful when compressed [2, 7].

In 2024, Torosis et al. published a consensus‐based treatment algorithm for HTPFD. Providers agreed that first‐line treatment for HTPFD should be pelvic floor physical therapy (PFPT); second‐line treatment includes trigger point injections (TPIs), vaginal muscle relaxants, and cognitive behavioral therapy; third‐line treatment consists of botulinum toxin injections; and sacral neuromodulation is a fourth‐line therapy. In this case series, we will focus on TPIs for the treatment of HTPFD, which are best used in conjunction with other therapies including PFPT in patients who have not seen adequate improvement with PFPT or who cannot tolerate or access therapy [2, 8].

TPIs are used to relieve myofascial pain via injection of trigger points [8]. Trigger points may be injected with a needle alone (dry needling), a local anesthetic with or without adding a corticosteroid, or botulinum toxin [8, 9]. At this time, there is no definitive evidence suggesting superiority of one injectate over the others [8, 10]. TPIs may also be performed with or without imaging guidance, such as fluoroscopy and ultrasound [8]. Studies have suggested that there are benefits to using ultrasound to guide injections including increased accuracy and avoidance of complications from blind injections [8, 11, 12]. TPIs targeting pelvic floor muscles can be external (pelvic) or internal (vaginal/transvaginal), and different approaches may be employed depending on the muscle groups being targeted. A transvaginal approach can be used in female patients to access deeper muscles such as the obturator internus (OI), obturator externus (OE), and levator ani (LA), while an external approach can be used to access superficial muscles including the gluteus, bulbospongiosus, and transverse perineal muscles [13–15]. Deeper muscles including the OI and LA can also be accessed externally via ultrasound guidance.

To the best of our knowledge, there has been no retrospective or prospective study that has comprehensively evaluated the use of external TPIs for pelvic floor dysfunction. Here, we present two cases where ultrasound‐guided external TPIs were successfully used to relieve myofascial pelvic pain due to HTPFD in female patients.

## 2. Cases

### 2.1. Patient 1

A 39‐year‐old woman presented to our pain clinic with myofascial pelvic pain due to HTPFD. The patient had a history of pelvic pain of multiple etiologies, including endometriosis, Crohn’s disease, and multiple abdominal surgeries. The pain was in the perineum with no radiation. The patient’s pain was worse with intercourse, straining, menstruation, and standing for long periods of time. The pain was made better by vaginal baclofen/diazepam suppositories, which the patient reported taking as needed for pain (about 10–15 times per year). The patient also noted that low‐dose naltrexone (3 mg daily) provided significant relief, and tizanidine (4 mg three times a day) helped somewhat. She had also trialed vulvar creams and a pudendal nerve block with no relief. The patient had also attended PFPT, and the PT evaluation confirmed inflammation around the pubic bone area.

External pelvic TPIs were performed in the superficial and deep transverse perineal muscles, pubococcygeus, iliococcygeus, coccygeus, and gluteus maximus muscles under ultrasound guidance using the steps discussed in the Procedure Description section that follows. The patient was feeling relief within 15 min, and she experienced good relief for several months. Six months later, the patient received an internal transvaginal TPI that did not provide relief. Two weeks after the unsuccessful internal TPI, the patient chose to repeat the external TPI and experienced good relief.

### 2.2. Patient 2

A 30‐year‐old woman presented to our clinic with myofascial pelvic pain due to HTPFD. The patient had a 15‐year history of pelvic pain of multiple etiologies including vaginismus and vulvodynia (s/p vulvectomy). The patient had a fall 20 years ago on her tailbone and had tailbone pain at the time of the visit. The pelvic pain was sharp in the perineal area and would shoot forward. The patient’s pain was worse with intercourse and menstruation. The pain was made better by consistent yoga practice and ibuprofen. The patient tried PFPT and biofeedback with no improvement. The patient also tried diazepam suppositories, which provided relief. She received a botulinum toxin injection 2 years prior to presentation to our clinic which provided some relief. She had a pudendal nerve block 1 year prior to presentation to our clinic, which provided 40% relief for 4–5 months.

External pelvic TPIs were performed in the bilateral gluteus, bulbospongiosus, and transverse perineal muscles under ultrasound guidance using the steps discussed in the Procedure Descriptio*n* section that follows. The patient was feeling relieved by the time the block had set. The TPIs were repeated twice more at 2‐week intervals for a total of three TPIs at each trigger point.

### 2.3. Procedure Description

**Figure 1:**
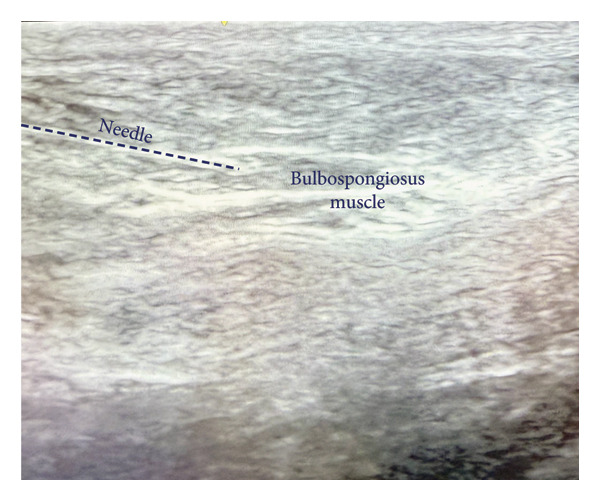
Ultrasound‐guided TPI of the bulbospongiosus muscle. *Source:* Alopi Patel, MD, 2025.

**Figure 2:**
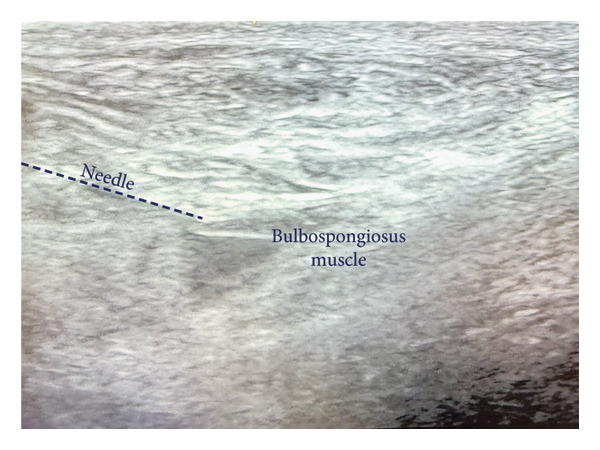
Ultrasound‐guided TPI of the bulbospongiosus muscle, alternate view.

The area over the myofascial spasm was prepped with alcohol using sterile technique. After isolating the area between two palpating fingertips, a 25‐gauge 3.8‐cm needle was placed in the center of the myofascial spasms, and a negative aspiration was performed under ultrasound guidance (Figures 1 and 2). Next, 1 cc of lidocaine 1% was injected into each trigger point bilaterally under ultrasound guidance in up to 10 locations.

## 3. Discussion

Both patients discussed in this case series showed multiple indications for treatment with TPIs, including distribution of pain consistent with the referral pattern of trigger points, restricted range of motion with increased sensitivity to stretch, focal tenderness, and a palpable taut band with reproduction of pain and spot tenderness along the length of the taut band with reduction of pain with pressure. As recommended in the HTPFD treatment algorithm published by Torosis et al., both of these patients used TPIs as part of a multifaceted treatment plan [2]. In our patients, these treatment plans included some combination of PFPT, suppositories, compound cream, oral medication, botulinum toxin injections, and pudendal nerve blocks.

Guidance suggests that repeat use of TPIs on a particular patient is only warranted if a patient responds to the initial TPI [2]. Based on our observations of Patient 1, who experienced no relief following a transvaginal TPI but had significant relief following an external TPI, we find it important to clarify that repeat TPIs using different approaches to target different muscle groups may be useful for HTPFD patients who have failed their first round of TPI therapy.

TPIs can be conducted with or without ultrasound guidance, based on factors including provider preference and the muscle group being targeted. It was previously thought that myofascial trigger points could not be identified using imaging, but recent studies have shown that ultrasonography can be used to accurately diagnose and visualize trigger points [11, 16]. Studies indicate that performing musculoskeletal injections in a number of anatomic locations under ultrasound guidance improves patient outcomes and safety when compared to approaches that do not use imaging guidance [8, 17]. For TPIs to treat myofascial pain specifically, there is evidence to suggest that ultrasound‐guided TPI techniques are more effective than blind TPIs, but research is limited on this topic [18]. Future studies are needed to compare the performance of TPIs with and without ultrasound guidance, specifically in the context of pelvic pain due to HTPFD in female patients. In this case, ultrasound guidance was used for the purpose of increasing safety by visualizing the anatomy to avoid accidental injury of surrounding structures. We advocate that all interventional pain physicians develop an understanding of pelvic musculature and be proficient in performing pelvic floor trigger point injections for patients with HTPFD, given the current shortage of physicians trained in this procedure.

## 4. Conclusion

After receiving ultrasound‐guided external TPIs, both patients in our case series experienced periods of relief. This suggests that ultrasound‐guided TPIs have a place as part of a comprehensive treatment program for patients with pelvic pain due to HTPFD and may be employed in cases where other treatment strategies have been unsuccessful. This case series highlights the need for additional research to evaluate the use of ultrasound‐guided external TPIs for the treatment of HTPFD.

## Ethics Statement

Ethics approval statement is not applicable.

## Consent

Patient informed consent was obtained for submission of a case report. The use of patient information in this case report is HIPAA‐compliant. None of the patients included in this case series was under the age to consent to participation.

## Conflicts of Interest

The authors declare no conflicts of interest.

## Author Contributions

Each of the authors has fulfilled all conditions for authorship including that they: (1) made a significant scientific contribution to the work, (2) are familiar with the content, and (3) are willing to take responsibility for the completeness and accuracy of the content.

## Funding

No funding was received for this study.

## Data Availability

Data sharing is not applicable to this article as no new data were created or analyzed in this study.

## References

[1] Speer L. M. , Mushkbar S. , and Erbele T. , Chronic Pelvic Pain in Women, American Family Physician. (2016) 93, no. 5, 380–387. 26926975

[2] Torosis M. , Carey E. , Christensen K. et al., A Treatment Algorithm for High-Tone Pelvic Floor Dysfunction, Obstetrics & Gynecology. (2024) 143, no. 4, 595–602, 10.1097/aog.0000000000005536. 38387036 PMC10953682

[3] Smith D. , Gugerty L. , Schug S. , and Lipetskaia L. , Triangulation of a Needs Assessment on High-Tone Pelvic Floor Dysfunction, Urogynecology. (2023) 30, no. 7, 622–627, 10.1097/spv.0000000000001435. 38031275

[4] Masi A. T. , Kamat S. , Gajdosik R. , Ahmad N. , and Aldag J. C. , Muscular Hypertonicity: a Suspected Contributor to Rheumatological Manifestations Observed in Ambulatory Practice, European Journal of Rheumatology. (2019) 2, no. 2, 66–72, 10.5152/eurjrheum.2015.0119. PMC5047265 27708929

[5] Simons G. D. and Mense S. , Understanding and Measurement of Muscle Tone as Related to Clinical Muscle Pain, Pain. (1998) 75, no. 1, 1–17, 10.1016/S0304-3959(97)00102-4, 2-s2.0-0031747078. 9539669

[6] Money S. , Pathophysiology of Trigger Points in Myofascial Pain Syndrome, Journal of Pain & Palliative Care Pharmacotherapy. (2017) 31, no. 2, 158–159, 10.1080/15360288.2017.1298688, 2-s2.0-85017115179. 28379050

[7] Lavelle E. D. , Lavelle W. , and Smith H. S. , Myofascial Trigger Points, Anesthesiology Clinics. (2007) 25, no. 4, 841–851, 10.1016/j.anclin.2007.07.003, 2-s2.0-36549006974. 18054148

[8] Srinivasan M. , Lam C. , Alm J. , and Chadwick A. L. , Trigger Point Injections, Physical Medicine and Rehabilitation Clinics of North America. (2022) 33, no. 2, 307–333, 10.1016/j.pmr.2022.01.011. 35526973 PMC9116734

[9] Chronic Pelvic Pain , Number 218, Obstetrics & Gynecology. (2020) 135, no. 3, e98–e109, 10.1097/aog.0000000000003716. 32080051

[10] Scott N. A. , Guo B. , Barton P. M. , and Gerwin R. D. , Trigger Point Injections for Chronic Non-malignant Musculoskeletal Pain: A Systematic Review, Pain Medicine. (2009) 10, no. 1, 54–69, 10.1111/j.1526-4637.2008.00526.x, 2-s2.0-59649110656. 18992040

[11] Sikdar S. , Shah J. P. , Gebreab T. et al., Novel Applications of Ultrasound Technology to Visualize and Characterize Myofascial Trigger Points and Surrounding Soft Tissue, Archives of Physical Medicine and Rehabilitation. (2009) 90, no. 11, 1829–1838, 10.1016/j.apmr.2009.04.015, 2-s2.0-70449331680. 19887205 PMC2774893

[12] Rha D. , Shin J. C. , Kim Y.-K. , Jung J. H. , Kim Y. U. , and Lee S. C. , Detecting Local Twitch Responses of Myofascial Trigger Points in the Lower-Back Muscles Using Ultrasonography, Archives of Physical Medicine and Rehabilitation. (2011) 92, no. 10, 1576–1580.e1, 10.1016/j.apmr.2011.05.005, 2-s2.0-81155162581. 21839982

[13] Bartley J. , Han E. , Gupta P. et al., Transvaginal Trigger Point Injections Improve Pain Scores in Women with Pelvic Floor Hypertonicity and Pelvic Pain Conditions, Female Pelvic Medicine & Reconstructive Surgery. (2019) 25, no. 5, 392–396, 10.1097/spv.0000000000000581, 2-s2.0-85059589825. 29621041

[14] Langford C. F. , Udvari Nagy S. , and Ghoniem G. M. , Levator Ani Trigger Point Injections: An Underutilized Treatment for Chronic Pelvic Pain, Neurourology and Urodynamics. (2007) 26, no. 1, 59–62, 10.1002/nau.20393, 2-s2.0-33846611867. 17195176

[15] Gupta P. , Ehlert M. , Sirls L. T. , and Peters K. , Transvaginal Pelvic Floor Muscle Injection Technique: A Cadaver Study, Female Pelvic Medicine & Reconstructive Surgery. (2017) 23, no. 1, 61–63, 10.1097/spv.0000000000000356, 2-s2.0-85010023354. 27898454

[16] Taheri N. , Okhovatian F. , Rezasoltani A. , Karami M. , Hosseini S. M. , and Mohammadi H. K. , Ultrasonography in Diagnosis of Myofascial Pain Syndrome and Reliability of Novel Ultrasonic Indexes of Upper Trapezius Muscle, Ortopedia Traumatologia Rehabilitacja. (2016) 18, no. 2, 149–154, 10.5604/15093492.1205022, 2-s2.0-84990932135. 28155823

[17] Daniels E. W. , Cole D. , Jacobs B. , and Phillips S. F. , Existing Evidence on Ultrasound-Guided Injections in Sports Medicine, Orthopaedic Journal of Sports Medicine. (2018) 6, no. 2, 10.1177/2325967118756576, 2-s2.0-85042874549. PMC5826008 29511701

[18] Kang J. J. , Kim J. , Park S. , Paek S. , Kim T. H. , and Kim D. K. , Feasibility of Ultrasound-Guided Trigger Point Injection in Patients with Myofascial Pain Syndrome, Health Care. (2019) 7, no. 4, 10.3390/healthcare7040118. PMC6956081 31618922

